# The first study of genetic diversity and population structure of Indo-Pacific bottlenose dolphin (*Tursiops aduncus*) and pantropical spotted dolphin (*Stenella attenuata*) in the Thai Andaman Sea based on ISSR

**DOI:** 10.14202/vetworld.2022.2004-2011

**Published:** 2022-08-22

**Authors:** Promporn Piboon, Anocha Poommouang, Kittisak Buddhachat, Patcharaporn Kaewmong, Kongkiat Kittiwattanawong, Korakot Nganvongpanit

**Affiliations:** 1 Department of Veterinary Biosciences and Public Health, Faculty of Veterinary Medicine, Chiang Mai University, Chiang Mai 50100, Thailand; 2 Excellence Center in Veterinary Bioscience, Chiang Mai 50100, Thailand; 3 Department of Biology, Faculty of Science, Naresuan University, Phitsanulok 65000, Thailand; 4 Phuket Marine Biological Center, Phuket 83000, Thailand

**Keywords:** cetaceans, diversity, Indo-Pacific bottlenose dolphin, pantropical spotted dolphin, population structure

## Abstract

**Background and Aim:**

The Indo-Pacific bottlenose dolphin, *Tursiops aduncus*, and the pantropical spotted dolphin, *Stenella attenuata*, are protected marine mammals in Thailand; however, knowledge regarding the populations of both species in Thai seas is minimal. We aimed to reveal the genetic diversity and population structure of two species, *T. aduncus*, and *S. attenuata*, based on inter-simple sequence repeats (ISSRs).

**Materials and Methods:**

Samples of stranded *T. aduncus* (n = 30) and *S. attenuata* (n = 23) found along Thai Andaman Sea coasts from 1998 to 2018 were used in this study. A total of 17 and 16 ISSR primers that produced clear and polymorphic bands were selected for *T. aduncus* and *S. attenuata*, respectively.

**Results:**

The highest percentages of polymorphic bands for *T. aduncus* and *S. attenuata* were 93.750% and 92.857%, respectively. Phylogenetic dendrograms indicated that the population of each species was clustered into three groups. This outcome was consistent with the genetic population structure, as both suggested three genetic clusters (DK = 3). Genetic diversity analysis revealed that the average Shannon’s information index (I) was 1.926 ± 0.066 for *T. aduncus* and 1.714 ± 0.090 for *S. attenuata*, which indicate a high level of genetic variation. Further, low fixation index (F) values were observed for *T. aduncus* and *S. attenuata* at −0.231 ± 0.024 and −0.312 ± 0.042, respectively, suggesting that inbreeding is unlikely to have occurred for both species over the past decades.

**Conclusion:**

At least three genetic clusters of both species were found in the Thai Andaman Sea, and the diversity indices of each species indicated that these species are not at a critical level for extinction. However, monitoring their population status should be prioritized to observe any future changes in the level of diversity.

## Introduction

All cetacean species inhabiting Thai seas, including two odontocetes identified as the Indo-Pacific bottlenose dolphin, *Tursiops aduncus*, and the pantropical spotted dolphin, S*tenella attenuata*, have been categorized as rare marine animals in Thailand. However, *T. aduncus* has been recognized as a common species found along the coasts of the Thai Andaman Sea and the Gulf of Thailand [1]. This coastal species is mainly found in shallow waters and exhibits a discontinuous distribution in warm temperate waters and those of the tropical Indo-Pacific Ocean [2, 3]. Meanwhile, *S. attenuata* is an oceanic species with a pantropical distribution in all oceans between 40°N and 40°S. Moreover, this species has been observed in marine waters along both coasts of Thailand [1, 2, 4]. According to the global conservation status assessment published in the International Union for Conservation of Nature Red List, *T. aduncus* was listed as a near-threatened species [3]. In contrast, the status of *S. attenuata* was of the least concern [5]. Furthermore, both species have been listed as protected animals in the Wild Animal Reservation and Protection Act of Thailand [6]; however, information regarding the population of these species in Thai seas is limited. In 2010, 80 *T. aduncus* and 90 *S. attenuata* individuals were reported to inhabit the Thai Andaman Sea [7]. All marine mammals worldwide, particularly small cetaceans, are currently impacted by various anthropogenic activities, which include commercial fisheries and other human interactions involving sea waters and generate oceanic pollution in the form of sea garbage and microplastics [8, 9]. The detrimental impacts of commercial fisheries include fisheries bycatch and environmental imbalances caused by prey depletion; these imbalances are associated with competition in the fishery industry [10, 11]. Fisheries bycatch is one of the greatest potential threats to small cetaceans worldwide [12–14]. *Tursiops* spp. and *S. attenuata* have been affected by various practices in the fishing industry in several regions of the world. Examples of these species include the offshore population of the common bottlenose dolphin *Tursiops truncatus*, which occurs in the Pilbara trawl fishery area in the waters of Northwest Australia [15]; *T. aduncus*, which is often caught using gillnet fisheries in the waters of Japan and Pakistan [16, 17]; and the population of *S. attenuata*, which is negatively impacted by the tuna purse-seine fishery in the waters of the eastern tropical Pacific Ocean [18, 19]. In the territorial waters of Thailand, fishery industries operate along both coasts of the country [20]. However, the number of dolphins annually affected by fisheries bycatch or commercial fishing gear remains unknown, particularly with respect to *T. aduncus* and *S. attenuata*.

Identification of management units is essential in developing conservation strategies for cetaceans, as most of these species are associated with extremely high mobility in marine environments, where geographical barriers are usually undetectable. Currently, genetic information is used to establish relevant management units for cetacean species in several regions of the world. In addition, this information is used in genetic population studies that have used nuclear and mitochondrial DNA markers for short-beaked common dolphins (*Delphinus delphis*) in Australian waters [21, 22] and the Irrawaddy dolphin (*Orcaella brevirostris*) inhabiting the Gulf of Thailand [23]. Due to the limited population studies over the past decades, there is scarce information regarding the populations of *T. aduncus* and *S. attenuata* inhabiting Thai seas. Consequently, the conservation status of the local population of these species in Thai seas cannot be evaluated. Thus, inadequate studies on local animal populations and inaccurate use of the conservation status could lead to ineffective management practices and may accelerate the extinction of local populations, as observed in the cases of the Antillean Manatee *Trichechus manatus manatus*, a subspecies occurring in Suriname [24], and the Songkhla Lake dolphin *O. brevirostris*, inhabiting the Songkhla Lake [12].

To monitor alterations in population diversity that may occur in the future and to collect additional information on the conservation biology of *T. aduncus* and *S. attenuata* in Thai seas, it is essential to acquire accurate genetic data. Thus, to the best of our knowledge, this is the first study to provide information on the genetic diversity and population structure of *T. aduncus* and *S. attenuata*, which inhabit Thai seas, based on inter-simple sequence repeats (ISSRs) using skin tissue samples from deceased dolphins.

## Materials and Methods

### Ethical approval

This study was approved by the Animal Use Committee of the Faculty of Veterinary Medicine, Chiang Mai University, Thailand, in 2020 (S14/2563).

### Study period and location

This study was conducted from June 2021 to February 2022 at the Faculty of Veterinary Medicine, Chiang Mai University, Thailand.

### Samples and DNA extraction

**Figure-1:**
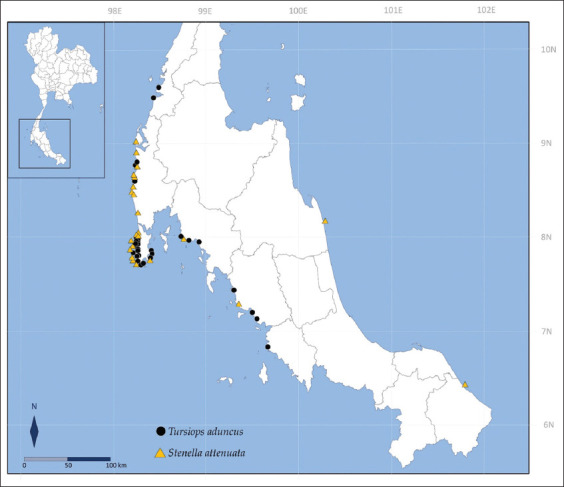
Map showing the location of stranded samples along Thai coasts during 1998–2018 [Source: https://www.rstudio.com/]. Black circles indicate *Tursiops aduncus*, and yellow triangles indicate *Stenella attenuata*.

The skin tissue samples of 30 deceased *T. aduncus* and 23 deceased *S. attenuata* dolphins, which were stranded along the Thai coasts from 1998–2018, were provided by the tissue bank of the Phuket Marine Biological Center, Phuket, Thailand (Figure-1). All samples were preserved in 95% ethanol at −20°C. DNA was extracted from these samples using DNA extraction kits according to the manufacturer’s instructions (DNeasy Blood and Tissue Kit, QIAGEN, Germany) at the Faculty of Veterinary Medicine, Chiang Mai University. The qualitative and quantitative analyses of the DNA samples were performed using agarose gel electrophoresis and spectrophotometry, respectively [25]. These samples were diluted to a final concentration of 10 ng/mL. Due to the poor quality of some of the DNA samples, 22 *T. aduncus* samples and 20 *S. attenuata* samples were used for subsequent analyses.

### ISSR amplification

A total of 34 ISSR primers from the University of British Columbia (Microsatellite UBC primer set 9, University of British Columbia, Vancouver, Canada) were initially screened to yield DNA fingerprints. Overall, 17 and 16 primers that produced reproducible bands were selected for the ISSR amplification of *T. aduncus* and *S. attenuata*, respectively; these amplified products were used for subsequent analyses (Table-1 for primer lists). Polymerase chain reaction (PCR) was conducted in 25 ml of reaction volumes containing 1× ViBuffer S (16 mM (NH_2_)_4_SO_4_, 50 mM Tris-HCl, 1.75 mM MgCl_2_, and 0.01% Triton™ X-100, New Jersey, USA), 0.2 mM dNTP (Vivantis, Selangor Darul Ehsan, Malaysia), 0.2 mM ISSR primer, 1 U *Taq* DNA polymerase (Vivantis), and 10 ng/ml of the DNA sample. The PCR amplifications were performed in PTC-200 using a DNA Engine Thermal Cycler (Bio-Rad Laboratories, Inc., CA, USA) under the following conditions: Pre-denaturation at 95°C for 5 min; 40 cycles comprising a denaturation step at 95°C for 30 s, annealing step at 55°C for 45 s, and extension step at 72°C for 1 min; and a final extension step at 72°C for 10 min. The PCR products were stained using RedSafe™ nucleic acid staining solution (iNtRON Biotechnology, Gyeonggi-do, South Korea). They were separated using 2% agarose gel (PanReac AppliChem ITW companies, Darmstadt, Germany) electrophoresis through a PowerPac 200 (Bio-Rad) containing 1× Tris-acetate-ethylenediaminetetraacetate buffer at 120 V for 30 min. Finally, the PCR products were visualized under ultraviolet light using a GelMax 125 Imager (UVP, Cambridge, England).

**Table-1:** The list of ISSR primers and the nucleotide sequences of ISSR primers used in this study, the number of total bands, polymorphic bands, and the percentage of polymorphic bands revealed by ISSR.

Species	Markers	Primer sequence	No. of total bands	No. of polymorphic bands	PPB
Indo-Pacific bottlenose dolphin (*Tursiops aduncus*)	UBC807	(AG)_8_T	8	3	37.500
	UBC808	(AG)_8_C	15	14	93.333
	UBC809	(AG)_8_G	11	9	81.818
	UBC811	(GA)_8_C	12	11	91.667
	UBC817	(CA)_8_A	13	12	92.308
	UBC818	(CA)_8_G	11	9	81.818
	UBC823	(TC)_8_C	9	8	88.889
	UBC825	(AC)_8_T	14	11	78.571
	UBC826	(AC)_8_C	14	13	92.857
	UBC827	(AC)_8_G	14	13	92.857
	UBC835	(AG)_8_YC	10	9	90.000
	UBC847	(CA)_8_RC	11	10	90.909
	UBC848	(CA)_8_RG	12	11	91.667
	UBC861	(ACC)_6_	16	15	93.750
	UBC874	(CCCT)_4_	15	13	86.667
	UBC880	(GGAGA)_3_	15	14	93.333
	UBC881	(GGGTG)_3_	13	10	76.923
	mean				85.580
Pantropical spotted dolphin (*Stenella attenuata*)	UBC807	(AG)_8_T	11	10	90.909
	UBC808	(AG)_8_C	9	8	88.889
	UBC809	(AG)_8_G	10	9	90.000
	UBC811	(GA)_8_C	9	8	88.889
	UBC817	(CA)_8_A	9	8	88.889
	UBC818	(CA)_8_G	12	11	91.667
	UBC825	(AC)_8_T	14	13	92.857
	UBC826	(AC)_8_C	11	10	90.909
	UBC827	(AC)_8_G	11	10	90.909
	UBC835	(AG)_8_YC	14	13	92.857
	UBC844	(CT)_8_RC	4	1	25.000
	UBC847	(CA)_8_RC	4	3	75.000
	UBC848	(CA)_8_RG	9	7	77.778
	UBC874	(CCCT)_4_	10	8	80.000
	UBC880	(GGAGA)_3_	10	7	70.000
	UBC881	(GGGTG)_3_	13	9	69.231
	Mean				81.486

PPB=Percentage of polymorphic bands, ISSR=Inter-simple sequence repeat

### Statistical analysis

The ISSR amplification results of the DNA samples from each species were analyzed separately. The bands or loci that were clearly observed and unambiguous among the ISSR amplified fragments were scored for the presence (1) or absence (0) of bands. A binary matrix was generated to determine the level of polymorphism in each primer, which was represented by the percentage of polymorphic bands calculated using the formula established by Ng and Tan [26] as follows:


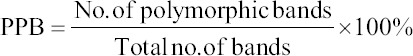


Phylogenetic dendrograms were then created using the DARWIN software version 6.0 (https://darwin.cirad.fr/) [27, 28] and Interactive Tree of Life web-based tools (https://itol.embl.de) such as clustering analysis. The groups obtained from this process were then used for genetic diversity and differentiation in population analysis.

### Genetic diversity and differentiation in populations

Genetic diversity parameters, including Shannon’s information index (I), observed heterozygosity (Ho), expected heterozygosity (He), and the fixation index (F), were determined using the GENALEX program, version 6.5 (https://biology-assets.anu.edu.au/GenAlEx/Welcome.html) [29]. Further, the genetic differentiation between the populations of each species was investigated using Nei’s genetic distance through GENALEX.

### Population structure

All samples of each species were analyzed using distance and model-based clustering methods to reveal population affinity and structure. The program STRUCTURE version 2.3.430 (Pritchard Lab, Stanford University, California, USA) [30, 31] was used to cluster individuals into populations based on ISSR genotypes, with assumed admixture and correlated allele frequencies. The LOCPRIOR model was used to infer cryptic population structure [32]. For both species, three runs for 1–10 clusters (K value) each were conducted to assess the presence of population structure with a Markov chain Monte Carlo burn-in length of 100,000 iterations and a run length of 1,000,000 iterations. Chain convergence was assessed by comparing the results obtained from three different chains. The ΔK statistics determined from the K values were then plotted using STRUCTURE HARVESTER (https://taylor0.biology.ucla.edu/structureHarvester/) [33] to identify the optimal number of clusters in the data. Outputs from the STRUCTURE HARVESTER were graphically modified using DISTRUCT (Rosenberg lab, Stanford University, California, USA) [34].

## Results

### ISSR polymorphism and phylogenetic dendrograms

**Figure-2:**
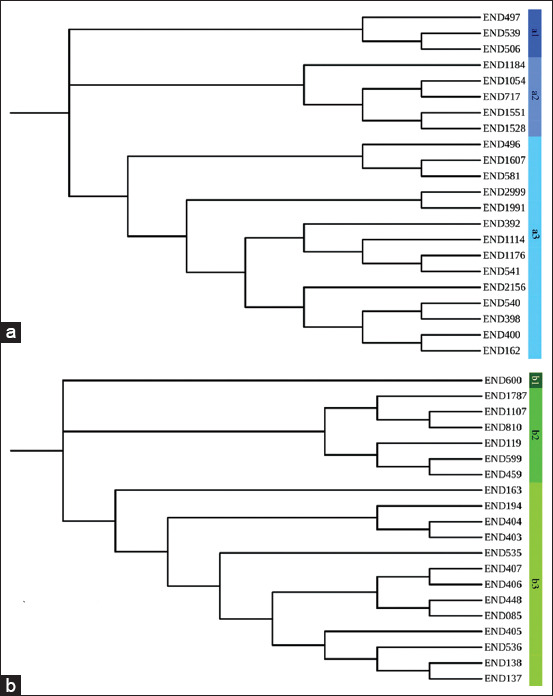
Phylogenetic dendrograms: (a) *Tursiops aduncus* and (b) *Stenella attenuata*.

Overall, 213 DNA fragments of *T. aduncus* were obtained using 17 ISSR primers (185 were polymorphic), whereas 160 DNA fragments of *S. attenuata* were obtained using 16 ISSR primers (135 were polymorphic) (Table-1). The highest and the lowest percentages of polymorphic bands for *T. aduncus* were 93.570% (UBC861) and 37.500% (UBC807), respectively (Table-1). In addition, the primers UBC825 and UBC835 generated the highest percentage of polymorphic bands for *S. attenuata* at 92.867%, whereas the primer UBC844 generated the lowest percentage of polymorphic bands at 25.000% (Table-1). Phylogenetic dendrograms of both species of dolphins are shown in Figures-2a and b. Each species was clustered into three main groups.

### Genetic diversity and differentiation

All values (I, Ho, and He) of *T. aduncus* indicated higher degrees of genetic variation in the a3 group than in the other groups (Table-2). Similarly, *S. attenuata* b2 group showed the highest genetic diversity for all indicators (I, Ho, and He). In the overall analysis, the Ho, He, and F of *T. aduncus* were 1.000, 0.817, and −0.231, respectively (Table-2). Meanwhile, *S. attenuata* showed Ho, He, and F values of 1.000, 0.772, and −0.312, respectively (Table-2). For *T. aduncus*, the results of pairwise Nei’s genetic distance analysis revealed that the lowest distance was present between the a2 and a3 groups (0.072). In contrast, the greatest distance was observed between the a1 and a3 groups (0.103) (Table-3). For *S. attenuata*, the results of pairwise Nei’s genetic distance analysis indicated that the closest relationship was between the b2 and b3 groups (0.110), and the most distant relationship was observed between the b1 and b3 groups (0.305) (Table-3).

**Table-2:** Diversity index values (mean±SE) for ISSR markers evaluated on *Tursiops aduncus* and *Stenella attenuata*.

Species	Group	I ± SE	H_0_ ± SE	H_e_ ± SE	F ± SE
Indo-Pacific bottlenose dolphin (*Tursiops aduncus*)	a1	0.974 ± 0.062	1.000 ± 0.000	0.598 ± 0.021	−0.705 ± 0.059
	a2	1.206 ± 0.079	1.000 ± 0.000	0.661 ± 0.024	−0.544 ± 0.058
	a3	1.912 ± 0.083	1.000 ± 0.000	0.823 ± 0.018	−0.227 ± 0.032
	All	1.926 ± 0.066	1.000 ± 0.000	0.817 ± 0.014	−0.231 ± 0.024
Pantropical spotted dolphin (*Stenella attenuata*)	b1	0.477 ± 0.083	0.688 ± 0.120	0.344 ± 0.060	−1.000 ± 0.000
	b2	1.464 ± 0.069	1.000 ± 0.000	0.725 ± 0.018	−0.394 ± 0.037
	b3	1.321 ± 0.136	0.938 ± 0.063	0.661 ± 0.052	−0.459 ± 0.068
	All	1.714 ± 0.090	1.000 ± 0.000	0.772 ± 0.022	−0.312 ± 0.042

I=Shannon’s information index, Ho=Observed heterozygosity, He=Expected heterozygosity, F=Fixation index, ISSR=Inter-simple sequence repeat, SE=Standard error

**Table-3:** Pairwise population matrix of Nei’s genetic distance of *Tursiops aduncus* and *Stenella attenuata*.

	Group a1	Group a2	Group a3	
Indo-Pacific bottlenose dolphin (*Tursiops aduncus*)	0	—	—	Group a1
	0.094	0	—	Group a2
	0.103	0.072	0	Group a3

	**Group b1**	**Group b2**	**Group b3**	

Pantropical spotted dolphin (*Stenella attenuata*)	0	—	—	Group b1
	0.286	0	—	Group b2
	0.305	0.11	0	Group b3

### Population structure

**Figure-3:**
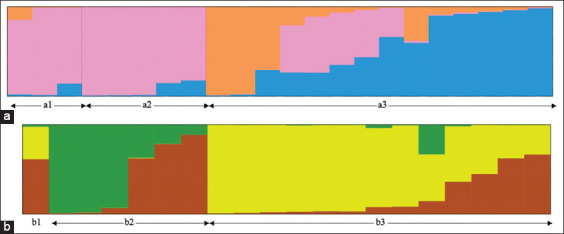
Admixture bar plot estimation of the dataset. The population structure consisted of three genetic clusters for both species indicated by different colors: (a) Orange, pink, and blue for *Tursiops aduncus*; (b) green, yellow, and brown for *Stenella attenuata*. Each individual specimen of each species is represented by a thin vertical line, which is separated into ΔK colored segments to represent the individual’s estimated membership fractions in each of the ΔK segments.

The results from three independent runs indicated that the population structure of both species comprised three genetic clusters (ΔK = 3). The a1 and a2 groups of *T. aduncus* had a dominant genetic cluster (Figure-3a). The b2 and b3 groups of *S. attenuata* represented two dominant genetic clusters (Figure-3b).

## Discussion

The results of this study provide genetic information on the population structure and genetic diversity of *T. aduncus* and *S. attenuata* inhabiting Thai seas. To the best of our knowledge, this is the first study to analyze the available skin tissue samples of two stranded species of dolphins along the coasts of Thailand. Three genetic clusters of both species were detected using population structure analysis. Although the diversity indices of each species revealed high genetic variation, further genetic studies using on-field or biopsy samples are needed to obtain the exact location of each population.

In this study, we found that the UBC primer set 9 produced DNA fingerprints that could be used to investigate the genetic diversity of *T. aduncus* and *S. attenuata*. The average percentages of the polymorphic bands for *T. aduncus* and *S. attenuata* were high at 85.58% and 81.48%, respectively. These values were higher than those of several land mammals, such as cattle, goats, sheep, and Asian elephants, which were included in the previous studies [27, 35]. These UBC primers could be used to effectively obtain DNA fingerprints used in previous genetic diversity studies for numerous plants, such as bryophytes [36] and rhubarb [37], as well as for animals, such as the Asian elephant [27], dugong [38], grass carp [39], porpoises [40], and *Limnonectes* [41].

According to the results of our study, the population structures of both species indicate that at least three genetic clusters of dolphin populations live along the Andaman coast. The Groups a1 and a3 of *T. aduncus* exhibited greater genetic relatedness to each other than to Group a2. This may be due to the fact that Groups a1 and a3 are likely to have a common ancestor in their recent evolutionary history. Similarly, according to *S. attenuata*, Groups b1 and b3 were more genetically related to each other than Group b2. Only one sample of *S. attenuata* obtained from the Gulf of Thailand (END194) was included in the population structure analysis, which revealed that the sample had not been separated from the other samples obtained from the Thai Andaman Sea and was determined to belong to Group b2. These results suggest a connection between *S. attenuata* populations inhabiting the Thai Andaman Sea and those inhabiting the Gulf of Thailand, as this oceanic species is also found around the Malay Peninsula, including the Malacca straits and in the waters of Singapore [5, 42]. Importantly, genetic information on these neighboring populations has not yet been elucidated.

The numbers of genetic clusters for dolphin populations differ in various regions because differences in marine environmental conditions can influence the diversification of each population. These conditions include water depth, ocean currents, sea surface temperatures, and the recycling of biological materials [43–45]. The three genetic clusters of the population of *T. aduncus* inhabiting the Thai Andaman Sea that were observed in our study differed from those of the previous studies conducted by Natoli *et al*. [46], which reported only one genetic cluster for a population inhabiting the KwaZulu-Natal coast of South Africa. In addition, Kiszka *et al*. [47] found that the population of *T. aduncus* in regions around Mayotte and adjacent shallow reef banks had at least two genetic clusters. Further, *S. attenuata* population inhabiting the Thai seas was categorized into three genetic clusters. This outcome is similar to that of the three genetic clusters of *S. attenuata* reported in a study conducted in Hawaiian waters by Courbis *et al*. [48].

A negative F value for both dolphin species in our study indicated high heterozygosity levels; thus, inbreeding is unlikely to be a problem for these dolphin populations. Both populations are unlikely to become extinct in the Thai Andaman Sea within a short period. Although the genetic diversity indices obtained in this study indicated a high level of variability, further genetic studies should be performed by including more samples. These studies should also include on-field or biopsy samples because using carcass-based studies to analyze population genetic structure can underestimate the genetic diversity in populations [49]. Importantly, genetic studies on the dolphin populations that inhabit the Gulf of Thailand would be precious. At present, the conservation status of the local populations of these two species inhabiting Thai seas is challenging to assess because of the scarce information on the abundance, core habitat, specific threats, and the number of dead animals of these species over the past decades.

## Conclusion

This study showed that ISSR can be used to successfully determine population structures and analyze the genetic diversity of *T. aduncus* and *S. attenuata* populations in the Thai Andaman Sea. At least three genetic clusters of both species were found, and the diversity indices of each species indicated that they are not at a critical level for extinction. However, due to the inadequate information on these populations and without proper monitoring plans, the abundance and genetic variability of these marine mammals may be significantly reduced in the near future. Thus, future studies are needed to address the existing knowledge gaps and to establish conservation plans for these populations.

## Authors’ Contributions

PP: Conducted the study, analyzed the data, and wrote the manuscript. AP: Conducted the study and analyzed the data. KB: Supervised the laboratory work and wrote the manuscript. PK and KK: Provided the samples and relevant data. KN: Designed the study, analyzed the data, and wrote and finalized the manuscript. All authors have read and approved the final manuscript.
